# Readability Gaps for Youth in Reproductive Health Materials: Comparing LGBTQ+ and Mental Health Content

**DOI:** 10.1192/j.eurpsy.2026.11938

**Published:** 2026-07-31

**Authors:** C. Pham, C. Noureddine, S. Simpson, C. Ramos, J. Toribio

**Affiliations:** 1 Icahn School of Medicine at Mount Sinai, New York; 2 Rutgers New Jersey Medical School; 3 Rutgers New Jersey Medical School, newark, United States

## Abstract

**Introduction:**

Health literacy is critical to equitable reproductive healthcare access, especially for adolescents and their families. Given that children and adolescents increasingly turn to the internet as their first source of health information—especially when stigma or fear complicate communication with adults—ensuring readability is crucial for early engagement and comprehension.

**Objectives:**

This study compares the readability of online reproductive health materials, focusing on general content versus those tailored for LGBTQ+ and mental health contexts.

**Methods:**

We evaluated the top 20 Google search results for: Reproductive Health vs. Mental Health, LGBTQ Health vs. Reproductive Health, and Queer Reproductive Health vs. Mental Health. 120 search results were analyzed using **WebFX’s Readability Tool**, which applies validated metrics including the Flesch-Kincaid Reading Ease. Texts were input directly without editing.

**Results:**

Readability varied significantly across topics (ANOVA, p < 0.001). Reproductive Health texts were the most accessible (mean Flesch-Kincaid Grade Level = 8.9 ± 1.8), while LGBTQ Reproductive Mental Health texts were the most complex (mean = 11.2 ± 5.1; p = 0.003 vs. Reproductive Health). Queer Reproductive Mental Health scores exhibited bimodal distribution, with both simple and complex outliers.

Complexity Hierarchy:

Reproductive Health < Reproductive Mental Health, p = 0.02

General Reproductive Health < LGBTQ/Queer Reproductive Health, p = 0.01

Mental Health-focused texts were consistently more complex than non-mental health texts, p< 0.05

**Conclusions:**

Hard-to-read LGBTQ+ and mental health materials reinforce existing barriers to care for marginalized identities. For younger individuals navigating sensitive topics like mental or reproductive health, accessible online materials can reduce stigma, empower autonomy, and serve as a critical entry point to care. Clearer online content is essential for advancing health equity.

**Disclosure of Interest:**

None Declared

